# "The Hospital" Nursing Mirror

**Published:** 1897-11-13

**Authors:** 


### "The Hospital" Nursing Mirror.

Being the Nursing Section of "The Hospital."

[Contributions for this Section of "The Hospital" should be addressed to the Editor, The Hospital, 28 & 29, Southampton Street, Strand, London, W.C., and should have the word "Nursing" plainly written in left-hand top corner of the envelope.]

### News from the Nursing World.

### QUEEN VICTORIA JUBILEE INSTITUTE.

It was announced at a committee meeting of the Queen's Commemoration Fund, held at Grosvenor House, that the fund on behalf of the Queen's Jubilee Institute had reached the splendid total of £70,000.

### AN EXPERIMENT IN DISTRICT NURSING.

An experiment in district nursing is about to be tried at Plaistow which will doubtless be watched with much interest by all who have the welfare of nurses and the sick poor at heart. Advocates of the cheaper systems, more especially the Holt-Ockley, of district nursing claim, with some show of reason, that the time of a highly-trained woman is too valuable to be spent on domestic duties such as a maid-of-all-work can easily discharge, and therefore seek to remedy the disadvantage by teaching the maid-of-all-work a modicum of nursing. This system is erroneous in principle and dangerous in practice. The new effort to improve matters is based on common-sense experiences, and promises well. The idea is to attach to each trained district nurse two attendants, who will, under her directions, perform all menial duties, whilst she confines herself to the strictly nursing tasks. These henchwomen will not be allowed to style themselves "nurse," and will be dressed quietly, but not in nurse's uniform. This method has certainly several points to recommend it to the attention of nurses and the public. In the first place women who are not fully qualified will not be called "nurse"; secondly, the sick will have the skilled attention that they need; and thirdly, the domestic duties that cannot be neglected will be performed by suitable women, and the nurse proper will consequently be free to attend to a larger number of patients.

### CROYDON NURSING ASSOCIATION.

The following statement of the Croydon Nursing Association has been received from its president. After saying that Sir F. Edridge simply expressed his own views in the remarks which were quoted by us a few weeks ago, he sends us a statement of account, which shows that £289 has been paid to the common fund on behalf of the two district nurses during the last year. This, of course, leaves a large balance in favour of the district nurses against the private staff, and quite disproves our charge that the earnings of the private staff were exploited on behalf of the district nurse. There are two things, however, which ought to be explained. How did it happen that such misleading statements as those quoted by us were allowed to pass unchallenged at the time, and what becomes of the balance of the district nurses' fund; is it absorbed in the general fund, and is it held over for another year? Would it not prevent mistakes and misrepresentations if the accounts to the two staffs were kept distinct, and any balance that might accrue, either to the one or the other, should be applied to the advantage of the staff to whom it rightly belongs? That both staffs occupy the same institute is no hindrance, if accounts are strictly kept.

### CHILDREN'S FETE AT THE MANSION HOUSE.

On November 4th the Lady Mayoress gave a charming and most successful fête in aid of the "Society for the Prevention of Cruelty to Children." The society's work is and has been most valuable, and now that it has resolved to carry out the recommendations of Lord Herschell's report loyally, its old supporters will doubtless rally round it once more with many new recruits to swell their ranks. The Lady Mayoress's idea of bringing home to the minds and hearts of happy children the sorrows of those other little ones whose natural protectors are their tyrants, was good, and the manner in which it was carried out picturesque. Troops of prettily-dressed children from all parts of the country marched up the banqueting hall to the Lady Mayoress and placed purses of gold on a silver salver in front of her, Miss Faudel-Phillips lifting up those who could not reach it. After this ceremony refreshments and dancing became the order of the day, and when the money in the purses was counted it was found to reach the sum of £1,600.

### IPSWICH NURSES' HOME.

Trained nursing in Ipswich has entered on its twenty-third year's work. The value and importance of the nurses' labours can be estimated by the steadily-growing demand for them. To two nurses a third has been added, and it is desirable to add yet three more. So far, the financial support is adequate. The committee wishes to affiliate the home with the Queen's Institute, and bring up the standard of efficiency to the highest level. There is some difficulty in getting cottage helps. The Committee of Management is re-elected, and probably next year will see a larger staff at work.

### WINTER HOLIDAYS FOR OUR NURSES.

Thanks to the organisation of foreign travel, now brought to such perfection by professional and universal couriers like Messrs. Thomas Cook and Son, our nurses need never dread a winter holiday. Already the foretaste of wintry chills are filling the trains, and sending summer wanderers flitting homewards from mountain country and the sea. Already those fragile lives dependent for breath itself on balmy air and sunny skies are speeding southward to a land of summer; and nurses can count up their money and pick and choose a tour according to inclination and cost. For £10 10s. each they can visit Rome, spending a day at Genoa when outward bent, and at Turin on their return. They may join parties leaving on November 17th, December 1st, 8th, and 22nd, and for a slightly increased expenditure prolong their stay abroad. An extra £5 5s. takes the tourist through Genoa, Rome, Florence, and Milan, and brings her home via the Italian lakes, St. Gothard, and Lucerne. We have not space to give particulars of the tours arranged through the Riviera, by the Mediterranean, Adriatic, Pyrenees, Spain, and Portugal; for such, intending travellers must apply to Messrs. Cook and Son, Ludgate Circus, E.C.

### QUEEN VICTORIA NURSING INSTITUTE, READING.

THE new Nursing Home at Reading is at 16, The Forbury; £3,000 has been subscribed for an endowment, and promises for annual subscriptions have been received. The house, which has been fitted up with electric light, has been adapted for its new use. The Mayoress performed the opening ceremony, and the Bishop of Reading read the dedicatory prayer.

### THE FIRST CLOUD.

THE troubles of the Irish Guardians after having complied with the order of the Local Government Board in appointing trained nurses have only just begun—witness the case of County Roscommon. Miss Hatton, one of the two new nurses, informed the medical officer in writing that there was a lunatic of whom she was afraid in the female ward, in consequence of which she objected to enter it. She also stated that unless her assistant could have proper rest during the day she could not possibly allow her to sit up at night. The doctor pronounced the letter "objectionable," and the Guardians informed the nurse that she was there to obey orders, and not to give them; but as Miss Hatton relies on having the Local Government Board on her side in the matter of the night nurse, it does not seem at all improbable that the affair is only a preliminary skirmish. It is only fair to the nurse to remind guardians that lunatics are certainly out of place, if not dangerous, in a sick ward, and that night work entails rest during the day, therefore every nurse they get will probably be equally "objectionable" on these points.

### SUNDERLAND NURSES' HOME.

THE foundation-stone of the new nurses' home attached to the Sunderland Infirmary was laid by Lady Laing on November 2nd. The cost, it is expected, will be defrayed out of the infirmary's share of the Queen's Diamond Jubilee Fund. The ceremony was largely attended, and Lady Laing was presented with a bouquet and a framed photograph of the infirmary. The new home comprises two wings, joining at right angles, with the staircase rising to a central tower at the juncture. Corridors run the full length of each wing on the side next the street, so that the rooms overlook the infirmary grounds, and the greatest degree of quietude is thus secured to their tenants. Fourteen bedrooms, a large sitting-room, bath, linen, and box rooms, &c., will be provided. Messrs. Joseph Potts and Son are the architects.

### EAST-END PHILANTHROPY.—SMALL SETTLEMENTS.

SINCE the formation of Toynbee Hall, settlements have arisen north, south, and east, and of all sorts and kinds; some are chiefly educational, and resemble polytechnics; some are smaller, and work on social lines. Merely to live amongst the people and be friends with them, sharing their joys and sorrows, is work worth doing. Two of these lesser settlements lately started are Newman House and the Hoxton settlement. The first is in Southwark, and is for men—a journalist, a barrister, and a civil servant live there, and give their evenings to clubs for men and boys. The Hoxton settlement has only women as residents, though it has men amongst its associates. It runs a girls' club, and a childs' guild of play, and the members visit in the neighbourhood. Miss Honnor Morten was the first resident, and her nursing experience is at the service of those neighbours who need it. The club is managed by Miss Hellicar, and the guild of play by Miss Stronach. More members are wanted for this settlement, especially those who have had training in hospital, and who now have time, experience, and money to offer where they are badly needed. In New York there are several nurses' settlements—there is not one in London. Perhaps Hoxton might become one, if further help and funds were forthcoming.

### VICTORIA HOME FOR NURSES, SWANSEA.

THE Mayor of Swansea (Howel Watkins, Esq.) laid the foundation-stone of the new Nurses' Home which is to be the town's commemoration of this year's rejoicings. It is arranged to accommodate eighteen nurses, and will be brought as far as possible up to the latest standard of excellence. £826 has been subscribed, and efforts will be made to raise this sum to £1,000. The home and heating apparatus will cost £2,250, but the Guesdon bequest may be available for the rest.

### BLOOD POISONING.

ONCE more a sad fatality brings home with fresh force to all engaged in attending septic wounds the danger of the slightest scratch. Dorcas Benford, for the past 14 years superintendent nurse at Wakefield Workhouse Infirmary, accidentally scratched her finger at the conclusion of an operation with a pair of scissors which had been used by the surgeon. She suffered no inconvenience until the next day, when the doctor dressed it, and all apparently went well for eight days, when she became very ill, and died of blood poisoning on November 4th. A consultant was called in, but nothing availed to check the disease.

### BURSLEM NURSING HOME.

BURSLEM District Nursing Home was opened on the 4th inst. by the Duchess of Sutherland. The whole cost, £560, has been collected. The Duchess gave a little history of a "jewel, which was neither glass nor paste; a jewel, which was a very remarkable possession for one generation, viz, the scheme of district nurses for the poor." Her Grace traced the origin to the work of the Sisters St. Vincent de Paul, in the seventeenth century, gave a tribute of recognition to Florence Nightingale, and described the appointment of the first district nurse at Liverpool in 1859.

### NURSING CHIEFLY.

THE Guardians of the Exeter Workhouse advertised for an assistant nurse. One applicant appeared, and, after viewing the hospital, declined the appointment on the score that there was no nursing to be done; it seemed to her "all washing and keeping them clean." The chairman promptly responded that that was "nursing chiefly."

### SHORT ITEMS.

THE prosperous working of Thetford Nursing Association during the first year of its existence and a balance of £23 encourage the committee to continue the work for another year.—At the fifth annual report of the Chesham Nursing Association it was announced that last June Miss Kiddle resigned in order to take more important work, and her place was taken by Miss Kelsey. The finances of the association are sufficient. —The district work at Rugby is outgrowing the strength of one nurse. There are no very poor people, but when sickness comes they are glad of the nurse's help. It seems a suitable neighbourhood to give a "provident basis" scheme a fair trial.

### Lectures to Surgical Nurses.

By H. A. Latimer, M.D. (Dunelm), M.R.C.S. of Eng., L.S.A. of London, Consulting Surgeon, Swansea Hospital; President of the Swansea Medical Society; Lecturer and Examiner of the St. John Ambulance Association, &c.

### XVIII. — DELIRIUM TREMENS: ITS NATURE, SYMPTOMS, AND TREATMENT BY THE NURSE —TREATMENT OF RIGORS AND OF THE "TYPHOID" STATE — THE BONES: THEIR USES IN THE BODY.

You may be called upon at any time to attend on a patient who becomes affected by delirium tremens—that special variety of delirium which attacks people who have drunk alcohol to excess; for it is a fact that when such people are stricken down by an accident or illness, which has lowered them, they are liable, quite suddenly, to suffer from this complaint. It is right, then, that you should know what symptoms arise, what is to be guarded against, and how you are to deal with this variety of delirium. The name given to the two most prominent symptoms which mark it is to some extent indicative of what occurs; rendered into English it means "trembling delirium." The sufferer talks to himself, and trembles in his limbs. He is affected by delusions and hallucinations, and is always in a state of great alarm and cowardice,—things not to be wondered at when we remember that these delusions and illusions take the form of snakes, cats, and scorpions, running over the bed or advancing towards him, or of fiends in the room. He sweats violently, suffers much from thirst, and is constipated; and, above all, is deprived more or less completely of sleep. He is liable to do an injury to himself, and is particularly apt to try to jump out of the window of his room; but he is not at all likely to do his attendants any harm, other than by struggling to get away from them. No doubt he makes for the window in a frantic effort to escape some one or more of the phantasms which are alarming him so intensely; and if he commit suicide, as he is often prompted to do, it is with the same view. The mere mention of the peculiarities of the complaint furnish you with some ideas of the precautionary measures which have to be adopted in dealing with it. All dangerous weapons or instruments, such as revolvers, knives, and razors, must be removed from the room; the window must be kept bolted, or a nail may be driven firmly into the frame above the bottom sash, so that it may not be suddenly thrown up; and constant, unremitting watchfulness must be maintained until the attack has yielded to treatment. It is astonishing how often these people jump out of windows, and either suffer some great injury or death in consequence. I presume it is because the watchers, worn out by long attendance, fall asleep, and then the madman, taking the opportunity so afforded him, or tortured by some sudden new delusion, makes a frantic rush to the nearest point of escape from the room. The treatment of these patients is by forcing them to take an abundance of nourishing food, in the form of broths, and eggs and custard, &c., and by the induction of sleep by drugs given under medical advice. As a rule, men attendants are placed in charge as the maniacal patients are strong when making their attempts to escape, and so require strength in opposition to their own, but now and then nurses of your sex are in charge of them. I say nothing to you about the question of whether or not stimulants shall be given; nor do I propose to instruct you as to the way to feed such patients by force and with the aid of the stomach tube—a measure which has to be adopted sometimes in extreme cases. But I will warn you, before I pass from the subject, that sufferers from delirium tremens sometimes die quite suddenly from failure of the heart's action, and that for this reason, in addition to the other ones I have given you, you should not be left alone with them until the attack has yielded to treatment.

At the onset, or during the progress of the surgical fevers of which I have been speaking, chills, or rigors, may take place. Treat them by applying warm bottles to the feet, and put some additional wraps on the bed; also give some warm drink—a hot cup of tea is as good as anything, or a teaspoonful of Liebig's extract of beef (which is an excellent stimulant), in a cup full of hot water. When the shivering attacks have passed you may be compelled to remove some of the bed-clothing again, and you will act as common sense directs you in this matter.

Now all that remains is for me to tell you what has to be done when the "typhoid" state declares itself, and, having told you much already about the general nursing of fever cases, there is very little more to be said on this subject. Not but that you will have plenty to do in such cases, but the plenty is summed up in a few words. Your great care will be to feed the patient at frequent intervals with nourishing food, and to administer the stimulants which will be ordered; to attend to the mouth and tongue, so that these foods may be swallowed with as little difficulty as is possible; to watch that the bladder does not become distended and to warn the medical attendant that the patient has not lately voided urine, should such be the case; and, above all, to prevent the formation of bed-sores. How you shall do this, and what your treatment of bed-sores will be, I will tell you when I come to deal with the subject of ulcers, of which they are one variety.

I propose now to treat on the subject of broken bones; and, judging from the fact that many accidents involving these structures occur, and that a good many of our beds here are occupied by sufferers from the same, I think you cannot fail to prove interested by what I shall have to tell you about them. I will commence by saying something about the functions of the bones, and their relative conditions of strength and elasticity at different times in our lives. With regard to the first point, I draw your attention to these facts: That they form the scaffolding of solid material on which our bodies are built up, and by the surfaces which they afford for the attachment of muscles, they enable these bands to move limbs. Let me illustrate this fact in a simple way by bending my fore-arm upon my arm. Notice that when I do this you can feel a fleshy lump on the front surface of my arm. The lump you feel is a muscle in a state of contraction. Being fixed to bones at the shoulder and fore-arm, by the tendons, it stretches as a band between these points, and when at rest is not to be particularly distinguished from the other muscles of the arm. But, having been ordered by me to bend the arm, it contracted or became shorter than it was, and, as it is fixed to bones above and below, those bones were compelled to draw nearer than they had been to each other, and the arm was "flexed." Apply this simple explanation to other movements of limbs, and you can see at once how important it is that we should have bones if we want to be able to move our bodies; and, furthermore, how important it is that our bones should be firm and unaffected by accident or disease. Another valuable purpose they serve is that of covering over and protecting organs which lie within them, as in the skull bones, which protect the brain from outside violence, and the ribs, which form a partial covering to the lungs and heart.

Thus when bones are broken or fractured, as is the usual mode of expression, movements of the parts with which they are connected are necessarily impaired, and organs protected by them are apt to be injured; so the subject is one which it behoves us to understand clearly with a view to our being able to remedy the resulting mischief.

### Post-Graduate Clinics for Nurses.

By a Trained Nurse.

### XXXV.—NURSING TYPHOID FEVER.

The widespread epidemic of typhoid fever at Maidstone gives an added importance to the subject of the nursing of this disease. I therefore propose to devote two or three clinics to some practical points with regard to the care and feeding of the typhoid patient in his several stages.

To be a "good typhoid nurse" is to have reached the Mecca of nursing, for to carry these cases through successfully argues much skill on the part of the attendant nurse. The first point to realise is the length and exhausting nature of the illness, and from the first hour to economise the patient's strength and keep him well fortified with food against the long siege.

We will take a supposititious private patient for one typical case—since the nurse in hospital must necessarily follow the traditions of the ward in which she nurses. I find the point on which so many nurses err is in nursing typhoid in rooms too cold. The window of the sick room should never be entirely closed, either by day or night, in the course of this disease, but considering the demands made on the vitality by the long-continued fever and the depressant nature of the illness, the patient cannot bear the further depressant of a cold room. A temperature of 58 deg. F. is commonly given as a suitable temperature for typhoid. Practically, we find patients at such a temperature need the bed full of hot-water bottles, and flannel-swathed legs, which probably means we are robbing them of heat by keeping them in a too cold room, and are paying back the heat loss by hot-water tins. Personally, I have found typhoid patients do infinitely better in a room kept uniformly at 63 deg.

A very distinguished medical man quite early in my nursing career, said: "I never allow my typhoid patients to use bed pans. So soon as I diagnose a case to be typhoid I instruct the nurses to immediately prepare fifty to sixty large pads of absorbent cotton wool sprinkled thickly with terebene powder, and enclosed in a covering of soft butter-cloth. These are slipped under the patient without any expenditure of his strength, and practically without movement. After use the nurse folds them in three and at once burns." In practice I have found this advice so good as to be beyond praise. In typhoid, as in so many other diseases, bed-sores are either caused, or, if begun, are increased by the use of the bed-pan, and the patient must necessarily be moved to a more or a less degree each time this is adjusted. By the use of these pads the rubbing of the back consequent on the use of the bed-pan is obviated, movement is minimised, and the danger to the community through typhoid excreta being introduced into the sewer system is prevented, for the pads are burnt. Any nurse who has once tried these absorbent pads will resort to them for other than typhoid patients. In those typhoid cases where the fæces and urine are passed unconsciously these pads are of the utmost value, for the constant changing of the bed linen which is necessitated in many typhoid cases where such pads are not used is most exhausting and harmful to the patient. This same doctor invariably instructs his nurses to use a plentiful sprinkling of terebene or ozone powder under the draw-sheet, and this pleasantly and hygienically obviates the unpleasant odour inseparable from typhoid cases. Another pet "wrinkle" of this physician—whose skill in typhoid is noteworthy—is to insist on the necessary abdominal binder being made from square sanitary towels, sewn together and fastened by tapes. This type of binder is lighter, more comfortable, and much less heating than when made from flannel. The typhoid patient should wear a thin, very loose Indian gauze long-sleeved vest. If it be large enough no inconvenience will result, and it may be quite short, so as not to come in the way of bed-pans, &c. One finds hospital-trained nurses so wedded to the idea of "a nightgown slit down the back" for typhoids and all other helpless cases, that it is difficult to dispel this tradition. But unless the patient requires frequent baths the thin light woollen gauze vest is a great advantage in typhoid; firstly, because the patient is so apt during his fever to throw off the bedclothes; secondly, because of the liability to bronchitis or pneumonia as a complication; thirdly, because of the open window which is necessary in this disease; and fourthly, if it be necessary to give as many headings as exist in a Scotch sermon, because of the alternating or remitted type of the typhoid temperature. A small fire should be kept in a typhoid room, for with fire and open window a very delightful atmosphere may be secured. The nurse must remember that the stupefied or comatose condition of the patient prevents him from complaining of chill or draughts, and that a room which to her robust health seems comfortable may be distinctly lowering to the vitality of the sick. A carpet should always be allowed in the typhoid room, for the quiet engendered of this will be soothing to the patient with delirium. The bad headache common in this disease should warn the nurse that the beds of such patients must never face the window, and in the headaching and delirious stage the room should be darkened.

In typhoid I like two narrow beds for the patient, one for the day and one for the night. Typhoids in India are always nursed thus, the heat of the climate making this plan essential. Certainly, in our temperate zone, the change of bed has a splendid effect in cases with high fever and delirium, and a fresh bed is an aid to sleep—and the sleeplessness of typhoid is a steady foe to a good convalescence. But the change must be effected ambulance fashion, by rolling two poles in the undersheet, and thus transferring the patient in a strictly horizontal position from one bed to the other. A single blanket is usually sufficient for warmth, with a clean sheet for counterpane, since counterpanes proper prove too heavy.

From the first day a square air or water bed cushion should be used, both for comfort and to obviate bed-sores.

In my nursing papers on typhoid I am not going to take up space by talking of the inflammation of "Peyer's patches." All this may be found in any nursing book, and most probationers can discourse learnedly on this distinctive feature of the disease. I am going to confine myself to the practical points of the nursing, and for my part I would rather have a nurse who could devise a delightful mouth-wash and bring me tempting dainty diet than one who could hold her own with a pathological professor on the histology of Peyer's patches. Only a week ago I saw at least fifty separate articles of clothing from typhoids, including blankets, sheets, and other valuable bedding, rent in rags and tatters after their disinfection by three-year certificated nurses, who had used crude carbolic acid. My own views are in favour of practical instruction, so that I shall leave the theory of the typhoid bacillus severely alone, and deal only with useful points.

### The Nursing of the Insane.

"The Nursing of the Insane and Epileptic" was the subject chosen for discussion on the afternoon of the second day's (October 27th) conference of the Union of Women Workers at Croydon, but being of far too wide a range to be dealt with in the short time set apart for the purpose, the first part—the nursing of the insane—alone was attempted. Miss Honnor Morten read a very able and interesting paper. After carefully guarding against any confusion between the treatment of the insane and the treatment of the epileptic, she rapidly sketched under three heads—the past, the present, the future—the bygone condition of things and the hoped-for condition in time to come. In the Middle Ages the lunatic was regarded as a wild beast and treated accordingly, being exhibited to the public for a fee of 2d. a head; more recently his treatment became rather that of a prisoner; and now, instead of being coerced and browbeaten, the poor gone-astray mind was taught by gentle persuasion and tact, and encouraged by surroundings and seeming liberty, to feel its way more and more to a firmer outlook. Great improvement began in the treatment of the insane with the founding at York, in 1792, of the Retreat, by William Tuke. In the thirties mechanical restraint was first set aside, later on, in the fifties, doctors suggested the training of attendants, and about twenty years afterwards schemes were formed for this purpose, notably that of Dr. Clouston, at Morningside, started in 1887. In 1891 the Medico-Psychological Association held its first examination for attendants on the insane. For this examination a two years' residence in an asylum was necessary. In some instances mental nurses were trained as nurses in a hospital, and this was being done with good results at the Northampton County Asylum. Here three years' training was required for a staff nurse's certificate, the examinations of the first two years being on general nursing, but that of the third year solely on mental nursing. From October, 1899, the Medico-Psychological Association would demand a term of three years' service before admitting candidates for examination, but should the candidate be a hospital-trained nurse the term would be reduced to two years.

The scheme suggested by Dr. Clouston was that the attendant be trained for two years in an asylum, and for one year in a hospital, the hospital training intervening between the two years' asylum training. This was then, at present, the outlook for the nursing of the mentally ill; the nurses would be trained by a mixture of hospital and asylum experience, by a series of lectures and classes, finally, examined, and, if proficient, certificated. Miss Honnor Morten concluded by referring to the Association of Asylum Workers, which numbers 2,668 members, and which aims at "raising the status and the training of asylum attendants."

### Presentations.

Nurse M. S. Matthewson has been presented with a handsome clock, as a token of love and esteem, from the matron and the nurses of the Royal Scottish Nursing Institution, Edinburgh, on the occasion of her marriage. She has nursed for 10 years in the R.S.N.I., and has been a great favourite with the doctors, surgeons, patients, as well as her fellow nurses, by whom she will be greatly missed. Her bright, genial manner always won the hearts of the sick and those whose privilege it has been to be her fellow workers.

### Minor Appointments.

Leamington Home for Incurables.—Miss Ella C. Cole, trained at the Royal United Hospital, Bath, was elected Assistant Matron of the above institution October 7th. Miss Cole has been night superintendent at Bath and has been in charge of Boxhill Infirmary Institute in affiliation with the Queen Victoria Jubilee Institute.

Blean Union Workhouse, Kent.—Miss Ellen Church, trained at Lambeth Infirmary, has been elected Head Nurse. She has formerly held appointments at the Royal Sea-Bathing Infirmary, Margate, and as staff nurse at Lambeth Infirmary.

Fulham Infirmary, Hammersmith.—Miss Jessie Bond Chambers, who was trained three years at St. Pancras Infirmary, Highgate, and was afterwards charge nurse, City of London Infirmary, is the new Charge Nurse of the above infirmary.

Union Workhouse, Lewisham.—Miss Clara Ann Curd has been elected Superintendent Nurse of the above workhouse. She was trained at Croydon Union Infirmary, and has been head nurse at Basingstoke Union Workhouse.

Carnarvonshire and Anglesey Infirmary, Bangor, North Wales.—Miss Constance Hulme, who was trained at this infirmary, has been appointed Charge Nurse of the women's wards.

The North-Eastern Fever Hospital, Tottenham, N.—Miss Agnes L. Potter has been appointed Charge Nurse at this institution. Miss Potter was trained at the Manchester Royal Infirmary.

City of London Infirmary.—Miss Henrietta Martin has been appointed Charge Nurse at the City of London Infirmary. She was trained at Mile End Infirmary, and has since held the position of staff nurse.

Newport Workhouse Infirmary.—Miss Jane Gillot, trained at Mill Road Infirmary, Liverpool, was elected Charge Nurse of the above institution on October 30th. She was formerly assistant nurse of Walton Union Infirmary.

Gravesend Milton Workhouse.—Miss Ann Clarke, trained one year at Birmingham and two years at St. George's-in-the-East, is the new Superintendent Nurse of this institution.

Farnham Union Infirmary.—Miss L. M. Graham was appointed Superintendent Nurse at the above infirmary on October 28th. Miss Graham was trained at the Ancoats Hospital, Manchester, and at the Clapham Maternity.

### Where to Go.

Queen's Hall, Langham Place, November 19th.—Concert by the Royal Amateur Orchestral Society, in aid of the special appeal fund for Charing Cross Hospital.

Midwives' Institute and Trained Nurses' Club, 12, Buckingham Street, Strand.—Dr. Boxall will lecture on "The Nursing of the Lying-in Woman," on Friday, November 26th, at a quarter to eight. Non-members can obtain tickets from the Secretary, price 6d.

### Wants and Workers.

The Boldmere District Nurse, Wylde Green, Birmingham, would be grateful for any old linen or flannel for a poor woman whose ailment necessitates a yard per day.

Nurse Gertrude would be so very grateful if someone would give a bed rest, air or water pillow or bed, bed-pan, feeding-cup, and spit-cup for the use of the sick poor in a large colliery district. If anyone can and will send either one or more of these things please send them to Sister Gertrude, Bagworth Station, near Leicester.

### The Gwelo Hospital.

Much interest was felt in the nursing world last spring when it became known that the Chartered Company had selected three English nurses to take charge of the new Gwelo Hospital, in Gwelo, Matabeleland.

The Company's hospitals hitherto had been under the care of Dominican nuns, and it was a new departure to appoint lay nurses. Miss Harding was selected as matron of the hospital, and the choice of her fellow nurses was put into her hands. She selected Miss H. Kenealy and Miss Muriel, of Norwich, and the trio started on their interesting and novel expedition on May 1st, prepared for many more difficulties and hardships by the way than they have been called upon to encounter. For their progress "up country," both by rail and mule waggon, was lightened by every kindliness on the part of the Company's officials and open-hearted hospitality from all with whom they came in contact. For a nursing uniform in South Africa seems to be the "open sesame" to kindness and pleasant experience.

It was a great surprise to the Sisters to find their new hospital so thoroughly up-to-date and modern in its methods, though the selection of the house and ward linen and some of the hospital furnishing was left to Miss Harding, whose skill as a colonial housekeeper rouses much local admiration. For the wardmaid and the scrubber are unknown in Matabele. All help is of a masculine nature, and it is not easy to the Matabele native to grasp the subtleties and niceties of the English conception of order.

The Sisters are delighted with their "home," which is a distinct building from the hospital, and consists of a bedroom for each and a nice large sitting-room, now being decorated by united effort into a very pretty and homelike apartment. It is an unusual luxury in Gwelo to have wooden floors and fireplaces. The Sisters rejoice in both, but a kitchen is not necessary, for all their food is sent from the hospital kitchen, and their needs excellently looked after by a man cook hailing from London. The ceilings are of canvas, and the luxury of doors with locks is much envied them by some of their neighbours, for locks in this part of the world are by no means a matter of course.

A certain bicycle case which protected a cherished machine on its long journey now forms a lovely wardrobe, and though upturned boxes once containing tins of a much-advertised milk still form the lockers of a few of the patients, all this will be remedied very shortly. And it is found that cane-bottomed chairs make excellent bedside tables until the looked-for arrival of the full contingent of hospital furniture. So few women patients ever come to hospital that there is no special ward for these, the one native ward being occupied by masculines. A Kaffir is at the head of this ward, being, of course, superintended by the Sisters and assisted by as many native "boys" as the heaviness and number of the cases warrants.

The utmost capacity of the hospital is accommodation for forty patients, but usually it is only during the spring "fever season" that there is so great a demand on the beds. A fever tent, which holds seven or eight patients, is specially put up for this busy season.

The patients consist chiefly of the members of the Mounted Police, of soldiers, and mining employés of the Chartered Company. But it is not necessary to be one of these in order to find a hospitable welcome and the best medical care possible.

Dr. Smythe, the doctor in charge, is a very busy man, for his practice extends to a sixty-mile radius of the hospital, and he is at all times ready at any hour of the day or night to set off cheerfully for a thirty-mile ride to attend an accident or see that sick or injured men are brought in to hospital in as comfortable a manner as possible.

Two efficient men orderlies, assisted by a sufficient number of Kaffirs, do the routine ward work under the Sisters' superintendence, one sister always being on night duty. Strange visitors prowl about the verandahs during the small hours—jackals and tiger-cats, and sometimes dark objects loom up in the distance which the orderlies and patients describe somewhat contemptuously as "only wolves." But to the London-trained nurse it is, to say the least of it, a little of a novelty to find wolves introduced into the landscape as seen from a ward window. But since lions are occasionally heard from the hospital grounds a mere wolf seems more or less of a trifle.

The hospital at Gwelo is built on a hill, and the air is extremely good and bracing, so that the patients suffering from malaria acquired in the neighbouring veldts at once feel the benefit of the high locality. Pneumonia and other lung diseases are common enough, and these are frequently complicated with malaria. Life in the mines is rough and hard, and the rest, quiet, and good food in the hospital has a wonderfully convalescent effect on the patients.

Two native women do the laundry work of the hospital, washing the clothes according to their custom, with feet instead of hands. It is extremely difficult to initiate them into the different uses to which different garments are put, so that they cannot appreciate that "Missie" prefers to have more care taken in the getting up of a hospital cap than is bestowed on a pair of stockings. Their own wardrobes are so extremely limited that an English outfit must appear astoundingly superfluous.

The conditions of hospital life at Gwelo are entirely different from anything that most nurses have experienced. The delightful hospitality of the people and the simple picturesqueness of life is charmingly restful. The only foes to combat are the white ants, who have a restless craving for the contents of feminine wardrobes; many varieties of ants abound, and cupboards with cake, jam, and other nice things destined to appear on sociable afternoons must needs be fitted with legs, and each of these disposed in a tin full of water, so as to depress the ant in his attempts to reach Excelsior!

One patient happened to put a few biscuits under his pillow, and his bed was speedily adorned with a string, several yards long, of industrious black ants, ready to dispute in uncomfortable fashion his monopoly of London-made biscuit—a novelty to the native insect.

The nursing sisters have been elected honorary members of the Gymkhana Club, where excellent tennis courts are provided, and occasional entertainments of an athletic nature are held. Ladies are somewhat scarce in Gwelo, and are welcomed in very charming fashion to the small community. In practical spirit the Sisters are each taking out a "claim" of land, to which they are entitled. "Taking out a claim" is accomplished at small cost, and such claim may turn out to be valuable, either through gold being found on it, or as a possible good building site.

In Gwelo there is a prejudice against women going out alone, especially after dusk, lest they "might meet natives." Most of these seem extremely harmless, but the prejudice is a remnant of the late war, the cruelties practised by the natives being of such a nature as not easily to be forgotten. And the Matabele are by no means to be trusted. But they make admirable patients, being uncomplaining, and able to bear any degree of pain, more especially of a surgical nature. Most nurses who have had experience of native nursing say they much prefer to nurse natives—especially the native women—to nursing Europeans. At first it is most difficult to judge of the condition of the patient, since the variations in skin colour are so difficult to distinguish. But time and experience teaches the nurse to easily translate the varying physical signs, just as they soon learn to recognise the different races.

The air of Gwelo is so good and pure that surgical cases do extremely well and rarely become septic. It would surprise many nurses from London to see the progress reached in the hospital there, and to find the up-to-date nursing appliances in use. The very newest drugs and dressings are used, and many nurses have declared that "the only properly ventilated hospitals they have ever been in exist in South Africa." The Chartered Company and their medical staff are certainly doing a splendid work in raising so high the standard of hospitals and reaching such a fine ideal of how the sick should be cared for. A new branch dispensary is just being established at Salonqui, which is about 25 miles from Gwelo. This will be under the superintendence of Dr. Smythe, whose zeal and enthusiasm for the welfare of the sick is untiring. Mr. Wright, the secretary and dispenser of the Gwelo Hospital, who did such splendid ambulance work during the late war, will be resident at Salonqui, and will be very much missed at Gwelo both by whites and natives alike.

### Everybody's Opinion.

[Correspondence on all subjects is invited, but we cannot in any way be responsible for the opinions expressed by our correspondents. No communication can be entertained if the name and address of the correspondent is not given, as a guarantee of good faith but not necessarily for publication, or unless one side of the paper only is written on.]

### HOUSEKEEPING.

"H. B." writes: If I am not troubling you too much I should like to know other district nurses' experiences of living on a salary of 16s. a week for everything except rent, coal, and uniform. I find living costs me one week with another 7s. per week eating and drinking only; that leaves me 9s. over for other housekeeping expenses firewood, oil for lamps, blacklead, boot blacking, &c., clothes, boots and shoes, travelling, charity, presents, luxuries, such as cakes or fruit, and last but not least illness and old age.

"A CONSTANT READER" writes: I shall feel grateful if some of your correspondents will give their opinion (through the medium of your valuable paper) as to the best and most economical way of keeping house on £40 a year. Imagine a district nurse to have £70 and an unfurnished house. She furnishes the house, and allows £20 a year for her holidays, clothes, and payments into Pension Fund, &c. So the £40 must find food, coals, lights, cleaning of her house, and wear and tear of utensils, and in fact general housekeeping expenses. She will be expected to work twelve hours (in her district) a-day, so will have little time for cooking or housekeeping. Of course, it can all be done easily by a good housekeeping management, so I shall be grateful to hear how from people of experience in the matter, of which I am ignorant.

### THE ROYAL BRITISH NURSES' ASSOCIATION.

NURSE M. E. GROOME, a member of the R.B.N.A., writes: If you have space in your valuable paper would you (if you think it worthy) insert this letter. I am a member of the Royal British Nurses' Association, and am really tired of hearing and reading what the public have to say against us respecting the so-called Rights Defence Committee. I and several of my fellow nurses express ourselves very sorry for Miss Foggo Thomson for the insults we consider she received at St. Martin's Town Hall on October 13th by the above-named committee. The meeting consisted chiefly of outsiders, people who knew absolutely nothing whatever about us, and had only heard one side of the question. I am perfectly certain our Royal President would not countenance anything that was wrong; therefore the question is who are we to be loyal to—Princess Christian or Mrs. Bedford Fenwick? I am perfectly certain we have the majority on our side, and that is for our Royal President. Even in this week's *Nursing Record* one who signs herself "Fair Play" says that matrons, sisters, and nurses should not tolerate petty tyranny and insulting patronage by a certain clique of medical busybodies, and, except in the sick-room, nurses are not dependent on the doctors. We know perfectly well, without "Fair Play" reminding us, that we are not depending on the doctors for our clothing, food, and wages, but to a certain extent we are depending on the doctors in private nursing; the doctors are our superior officers, it is our duty to obey them, and I certainly consider them more fitting to rule the Royal British Nurses' Association than a body of women. I cannot help thinking that "Fair Play" should mind her own business, and not interfere with matters that do not concern her.

### Irish Distressed Ladies' Fund.

### AN APPEAL.

SIR,—Last year you were good enough to insert in your columns an appeal from us on behalf the Irish Distressed Ladies' Fund. The causes of that distress are now so well known that it is hardly necessary for us to recapitulate them; suffice it to say that, although the ranks of those in need of relief have been thinned by death, many sad and distressing cases still remain, and but for the kind and charitable aid afforded by the public, many ladies formerly in good circumstances but now utterly destitute would ere this have been obliged to seek parochial relief if not to end their days in the workhouse. The London and Dublin committees have received during the year, including a legacy of nearly £400, the sum of £2,608, and they have expended £2,307. This expenditure includes £1,051 in pensions to 75 old ladies, £139 for education, £354 in special grants, and £34 for illness. Permit us to say how grateful our committees are to those who subscribed to the fund during the present year; they sincerely trust that they may be again liberally supported with subscriptions, in order that the good work which the charity has carried on for so many years may not be stopped through want of funds. The secretaries will thankfully receive and acknowledge subscriptions at 17, North Audley Street, London, and 4, Kildare Street, Dublin.—Your obedient servants,

ERNE, Chairman of the London Committee.
BELMORE, Chairman of the Irish Committee.

November 1st, 1897.

### The Royal British Nurses' Association.

WE are asked to publish the following statement: The attention of the Executive Committee has been called to a circular recently sent to a large number of nurses asking them to allow their names to be attached to a petition against the sanction of the new bye-laws by the Privy Council. The committee desire to point out to those nurse members who may have received the circular, that they are asked to authorise their names to be attached to a document they have never seen, and to petition against bye-laws which they have had no opportunity of reading or discussing. The circular also contains statements as to the nature of the bye-laws which are misleading and inaccurate. Nurses are strongly recommended to postpone any action in the matter until they have had an opportunity of discussing and considering the bye-laws at the general meeting, which will shortly be held, and which every nurse member will be invited to attend. Nurses who have signed the post-card sent to them under a misapprehension as to their nature or authority should at once let this be known to the Secretary of the Association, 17, Old Cavendish Street, W.

### For Reading to the Sick.

### THROUGH ALL GIVE THANKS.

**Verses.**

THOSE who in affliction strive to bless
A Father's rod, shall on His staff recline,
And in grief's darkest hour be cheer'd
By light divine. —*Bernard Barton.*

When all Thy mercies, O my God,
My rising soul surveys,
Transported with the view I'm lost
In wonder, love, and praise!
Ten thousand, thousand precious gifts
My daily thanks employ;
Nor is the least a cheerful heart
That tastes those gifts with joy—
Through all Eternity, to Thee
A joyful song I'll raise!
For, oh! Eternity's too short
To utter all the praise! —*Addison.*

O Lord of Heaven, and Earth, and Sea,
To Thee all praise and glory be!
How shall we show our love to Thee—
Giver of all?
For peaceful homes and healthy days,
For all the blessings earth displays,
We owe Thee thankfulness and praise,
Giver of all. —*C. Wordsworth.*

We thank Thee, then, O Father,
For all things bright and good,
The seed-time and the harvest,
Our life, our wealth, our food!
Accept the gifts we offer
For all Thy love imparts,
And—what Thou most desirest—
Our humble, thankful hearts.
All good gifts around us
Are sent from Heaven above!
Then thank the Lord, oh! thank the Lord,
For all His love. —*Claudius.*

"I have sinned," she said, "and not merited
The gift He gives, by the grace He sees!
The mine-cave praiseth the jewel, the hill-side praiseth the star!
I am viler than these!"
Then I cried aloud in my passion—"Unthankful and impotent creature,
To throw up thy scorn unto God through the rents in thy beggarly Nature,
If He, the all-giving and loving, is served so unduly—what then
Hast thou done to the weak and the false and the changing—thy fellows of men?" —*E. B. Browning.*

**Reading.**

Every creature of God is good, and nothing to be refused, if it be received with thanksgiving.—1 *Tim.* iv. 4.

In everything give thanks; for this is the will of God in Christ Jesus concerning you.—1 *Thess.* v. 11.

Each day brings its trials; but their bitterness passes away, while the sweetness of its blessings remain, like the rose-scent in the vase. The unnumbered kindnesses of God, the countless small pleasures which mark his unslumbering thoughtfulness for us, are like bright ears of corn scattered along our path, to tell us of the golden harvest field from whence they come and towards which we are journeying; crystal drops from the river of life, which maketh glad the city of God. Such things cheer us on in this earth, but should not bind us to it; rather should they send us onward with joyfulness, eager for the bliss that God has prepared for us on high, and longing for those pleasures which are at His right hand for evermore.

### Notes and Queries.

**The contents of the Editor's Letter-box have now reached such unwieldy proportions that it has become necessary to establish a hard and fast rule regarding Answers to Correspondents. In future, all questions requiring replies will continue to be answered in this column without any fee. If an answer is required by letter, a fee of half-a-crown must be enclosed with the note containing the enquiry. We are always pleased to help our numerous correspondents to the fullest extent, and we can trust them to sympathise in the overwhelming amount of writing which makes the new rules a necessity. Every communication must be accompanied by the writer's name and address, otherwise it will receive no attention.**

*A Short Nurse.*

(53) 1. I am anxious to be a trained nurse, but have been refused at several hospitals on account of my height (5 ft.). Will you kindly tell me if there are any hospitals or institutions where I could get the training with small salary? I am 23, and have been a lady nurse for several years. 2. I would like to know what is the charge for an advertisement in your paper.—*Nurse M.*

1. Most probably an advertisement will serve your purpose best. 2. About 1s. 6d.; it varies according to the length.

*Housekeeping.*

(54) Will you please inform me as to best course to take to qualify for matron? I am a three years' trained nurse, but have had no experience in housekeeping nor yet in general management, and those appear to be the leading qualifications. I shall be delighted if you can help me by your suggestions.—*Nomenclature.*

Try first for a post as assistant matron, where you will have opportunities of acquiring the knowledge you lack.

*Lectures.*

(55) I should feel greatly obliged if you could kindly tell me where I could get a course of simple lectures on ventilation, circulation, fevers, &c., to read to my nurses (five in number) during the winter months. They have most excellent ward work, both medical and surgical, but I feel sure a course of lectures would be most helpful.—*M. W. Deacon.*

"Humphrey's Manual of Nursing, Medical and Surgical," post free, 3s. 6d.; "Fever Nursing," William Harding, M.D.; "Elementary Physiology for Nurses." C. T. Marshall, M.D., F.R.C.S. The Scientific Press, 28 and 29, Southampton Street, Strand.

*Surgical Training.*

(56) Can you tell me of any private surgical home or hospital where I can obtain a year's training, either by receiving a small salary or giving my time? Also if there are any provincial hospitals where I might receive training for a year? I have learned midwifery, but cannot get enough work to keep me employed as a monthly nurse. Is there any institution one can join with only the midwifery certificate?—*A. M. Foster.*

1. An advertisement will help you best. 2. The Midwives' Institute, 12, Buckingham Street, Strand. You should be able to get work. Write first to the Midwives' Institute, and the secretary will tell you what advantages you obtain by becoming a member.

*Monthly Nursing.*

(57) Will you please tell me where a monthly nurse, certified only for same, could join to get work, as suggested in your "Notes and Queries" last week?—*Elizabeth.*

Watch our advertisement columns, and advertise yourself.

*Nursing.*

(58) I hold a certificate for children's nursing, but lack experience in the care of young infants. Would it be useful for me to enter my name at Queen Charlotte's? (2) How long would it be before I should be able to make my living by monthly nursing or midwifery? (3) Is the training expensive?—*Nita.*

It is best for you by far and away to train for the L.O.S. diploma. You can enter at Queen Charlotte's or at any maternity hospital you like, and take monthly nursing for three months, and then another three months for the L.O.S. When you have this diploma you can begin work as soon as you can get it. The fees are very moderate, considering that they secure you a profession for life. Monthly nursing at Queen Charlotte's costs £15 15s. for three months; midwifery, £25 guineas for three months. At the General Hospital, York Road, monthly nursing for three months, £10 10s.; if pupil continues for midwifery, the whole six months is £30. The cheapest is East London Mothers' Home. It is very good; the cost is about £15 15s. for the entire course.

*The Powers of Ladies' Committees.*

(59) When a district nurse furnishes her own rooms and pays half the rent, have the ladies on her committee any right to enter and examine her premises in her absence? In these circumstances is it fair on their part to send her a letter of censure because a boy of eight and a girl of ten were sleeping in the same room?—*Mary C.*

The ladies of the committee have every right to pay surprise visits, but it certainly would have been kinder to have chosen an hour when the mistress of the house was at home. With regard to the two children sleeping in the same room, you must see for yourself how very undesirable it is. It is one of the great evils of overcrowding, to which so much anxious thought is given, and a district nurse must be an example in all things relating to hygiene and morality. The gist of the whole trouble is that the salary paid is not enough to do everything, and perhaps if you represented this to your committee, and explained the difficulties you were put to in order to make both ends meet, they would be more considerate. We are making inquiries, and shall perhaps hear of a nurse to live with you as you want.

*Private Nurses' Home.*

"J. M." is reminded that we do not report on an institution except after personally inspecting it. The name and address is noted, so that, should a representative of THE HOSPITAL visit Margate, a notice can be written.

